# Scorecard Approach to Eliminate Onchocerciasis in Venezuela

**DOI:** 10.4269/ajtmh.23-0743

**Published:** 2024-06-11

**Authors:** Oneida Camacho, Carlos Botto, Dalila Ríos, Benny Barrios, Sharly Ceballos, Oscar Noya-Alarcón, Miguel Fernández, Yseliam Tovar, Nestor J. Villamizar, Lindsay Rakers, Frank Richards, María Eugenia Grillet

**Affiliations:** ^1^ Centro Amazónico de Investigación y Control de Enfermedades Tropicales “Simón Bolívar” (SACAICET), Ministerio del Poder Popular para la Salud, Puerto Ayacucho, Venezuela;; ^2^ Onchocerciasis Elimination Program for the Americas (OEPA), Carter Center Guatemala Office, Guatemala City, Guatemala;; ^3^ The Carter Center, Atlanta, Georgia;; ^4^ Laboratorio de Biología de Vectores y Parásitos, Instituto de Zoología y Ecología Tropical, Facultad de Ciencias, Universidad Central de Venezuela, Caracas, Venezuela

## Abstract

In the Americas, onchocerciasis has been eliminated in 11 of 13 endemic foci by mass administration of ivermectin. The remaining at-risk population resides in a contiguous cross-border transmission zone located in the Amazon jungle in northwest Brazil and southern Venezuela, known as the Yanomami Focus Area. Here, we describe the development and implementation of a data-driven tool, called the Scorecard Approach (SCA), for the 393 communities that comprise the Venezuela South Focus. The SCA was first applied in 2018 and is reassessed on an annual basis. This operational strategy seeks to prioritize communities with low ivermectin coverage while taking into account the nature and variation of other epidemiological and logistical variables. Numeric scores are assigned for each factor and added together to yield a composite score for each community that is categorized as high, medium, or low priority. In this way, the SCA serves as a valuable and comprehensive strategy for planning, monitoring, and maximizing programmatic efficiency. In addition, it has allowed the country to face the main challenges of this endemic area: its remoteness, its large areas of territory to cover, the semi-nomadic nature of the Yanomami people, and their continuous cross-border movements. For 2022, the SCA categorized 54 (13.7%), 108 (27.5%), and 231 (58.8%) communities as high, medium, and low priority, respectively. The results presented here show that prioritizing communities at risk and with greatest needs increases the feasibility of interrupting the transmission of onchocerciasis by 2025 in the last endemic focus in the Americas.

## INTRODUCTION

Human onchocerciasis is a chronic infection caused by the parasite *Onchocerca volvulus* and transmitted by the infective bites of vectors of the genus *Simulium* (blackflies). The embryonic stages of the parasite, microfilariae (mf), migrate through the skin and cause severe itching, skin disease, and eye lesions in the human host. The severity of the clinical manifestations depends on the mf density in the skin, which is in turn related to the intensity and duration of exposure to blackfly bites.^1^ A goal of onchocerciasis transmission elimination worldwide was recently established by the WHO,^2^ and the risk of blindness or skin disease related to onchocerciasis has been reduced in endemic areas by the mass drug administration (MDA) of ivermectin (Mectizan^®^, Merck & Co., Inc., Rahway, NJ). Ivermectin kills mf and temporarily inhibits their release by gravid adult female worms, as well as killing adult worms after several years of mass treatment given at 3–6-month intervals.^3^^,^^4^

In the Americas, the infection was formerly prevalent in six countries (Brazil, Colombia, Ecuador, Guatemala, Mexico, and Venezuela), where 538,517 persons were considered at risk of infection.^5^ In 1993, a regional initiative called the Onchocerciasis Elimination Program for the Americas (OEPA) was formed with a unified strategy to eliminate new (ocular) morbidity caused by *O. volvulus* and interrupt parasite transmission by delivering MDA twice a year to ≥85% of the eligible population in all endemic communities.^6^ The OEPA was initially supported by the River Blindness Foundation and from 1996 onward by The Carter Center. This regional initiative has been very successful, and the WHO has since verified the elimination of onchocerciasis transmission in Colombia (2013), Ecuador (2014), Mexico (2015), and Guatemala (2016). In Venezuela, the elimination of transmission has been attained for two of the three transmission zones (foci) in the country: Northcentral (2014) and Northeast (2017).^7^ Overall, the regional achievement represents a 93% reduction of the original at-risk population for onchocerciasis, with the remaining 7% (38,045 individuals) residing in the Yanomami Focus Area (YFA), a cross-border contiguous transmission zone composed of Brazil’s Amazonas Focus and Venezuela’s South Focus.^5^ The Venezuela South Focus is one of the smallest of the original foci in the Americas in terms of population (3.15%), but is the largest focus in the Americas by area (approximately 230,000 km^2^). Before the start of ivermectin MDA, the YFA had zones of high intensity of transmission and the significant presence of severe ocular lesions and blindness^8^^,^^9^; because of its remoteness, it has always been considered the most difficult area in the Americas in which to eliminate onchocerciasis transmission.^10^ The American region continues to target interruption of transmission of onchocerciasis by 2025, in accordance with the Pan American Health Organization.^11^

Transmission of onchocerciasis in the Venezuelan South Focus takes place in the rainforest of the Venezuelan Amazon region, affecting mostly the indigenous Yanomami, followed by the Yekwana and Hoti people.^9^ Three different *Simulium* species are found in the areas, from greatest to least vectorial capacity: *Simulium guianense* sensu lato (s.l.), *Simulium incrustatum*, and *Simulium oyapockense* s.l.^12^^–^^14^ A high biting density of *S. guianense* s.l. has been significantly associated with hyperendemic communities, but so have other variables such as landscape and sociocultural and environmental factors (e.g., topography, ethnic group, and river size). Specifically, the various forms of movement and displacement of the Yanomami population through different geographic areas and landscapes within the endemic area expose the human population to different risks of infection and provide an opportunity for transmission to persist. Consequently, the heterogeneity of onchocerciasis transmission in this endemic area is associated with the heterogeneity of the geography, the diversity and complexity of a multi-vector transmission system, and the use of tropical forest space and resources by Yanomami people.^9^

The OEPA initiative has demonstrated that sustaining high geographic and effective (≥85% coverage of eligible population) coverage for each treatment round is essential for interruption of onchocerciasis transmission.^6^ In 2000, a twice-per-year treatment strategy was initiated in the Venezuelan South Focus, and in 2009, treatment frequency was increased to four times per year in 80% of hyperendemic communities to hasten interruption of transmission in areas showing slow progress or in communities recently identified and incorporated into the program at later stages. Combined results from parasitological, entomological, and serological evaluations, as well as information on the total number of effective treatment rounds, showed that in 2016, onchocerciasis transmission and morbidity suppression had been reached in 75% of the Yanomami population and 70% of all known (mapped to that date) communities.^8^

Recent advances in the spatial epidemiology of the Yanomami onchocerciasis area in Brazil and Venezuela^9^ have allowed both elimination programs to devise new strategies together to maximize programmatic efficiencies to suppress the last sources of parasite transmission in the YFA and stop all MDA in accordance with the global objective established by the WHO.^2^ These strategies are designed to overcome the barriers of remoteness and large expanse of Yanomami territory to cover, the semi-nomadic characteristics of the human population, and continuous cross-border movements between Brazil and Venezuela.^9^ Here, we report and describe the development, adaptation, and implementation of a new strategy, the Scorecard Approach (SCA), which was designed to identify those communities with lagging progress in terms of ivermectin coverage and to orient and focus more effectively on the priorities for eliminating onchocerciasis in the Venezuelan South Focus.

## MATERIALS AND METHODS

### Study area.

**Figure 1.**
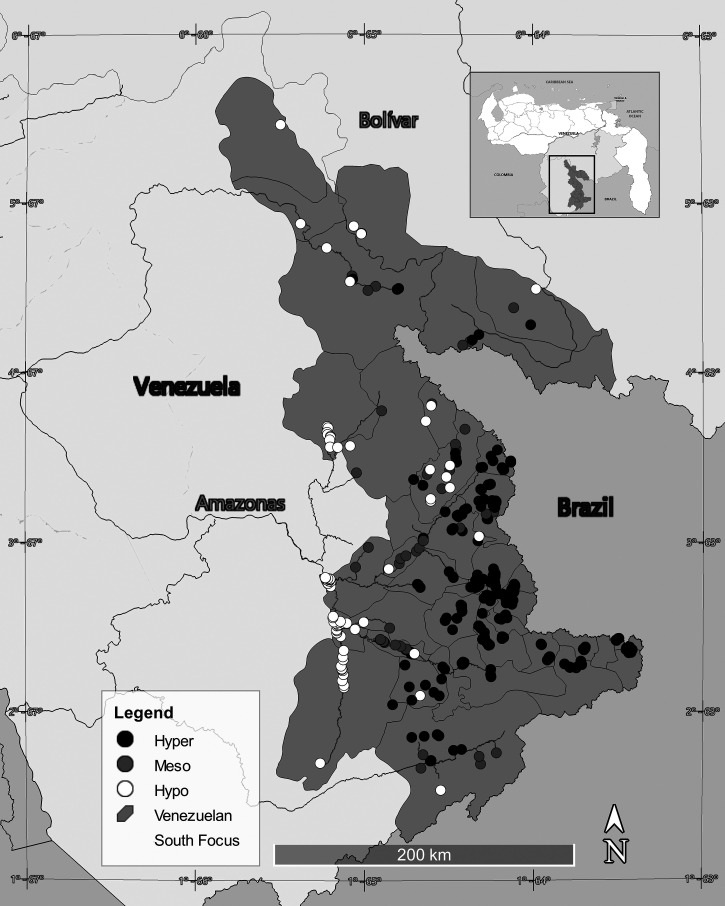
Geographical distribution of hypo-, meso-, and hyperendemic communities in the Venezuela South Focus as determined by the prevalence of *Onchocerca volvulus* microfilariae detected microscopically in superficial skin biopsies.

**Figure 2.**
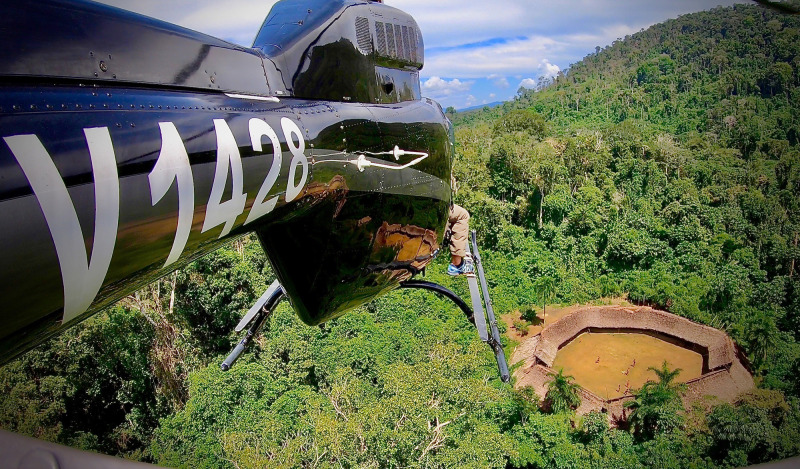
Yanomami *shapono* in the Venezuelan rainforest inhabited by Yanomami people and surrounded by a plantain garden as viewed from a Program helicopter (photo by O. Noya-Alarcón).

Onchocerciasis transmission in southern Venezuela occurs in the lowlands (0–500 m) and uplands (500–1,200 m) of the Amazonas and Bolívar states (Figure 1), specifically in the Upper Orinoco, Upper Ventuari, Upper Siapa, and Upper Caura river basins. The Yanomami at-risk population in Venezuela is largely semi-nomadic and is estimated currently as 18,118 individuals dwelling in approximately 393 “shaponos” scattered deep in the forest (Figure 2).

### Epidemiology of endemic communities.

Baseline endemicity was determined over the years based on the prevalence of *O. volvulus* mf detected and counted microscopically in two skin biopsies from the iliac crests (right and left) of sampled individuals.^8^ Communities were classified as hyperendemic (mf prevalence ≥60%), mesoendemic (mf prevalence ≥20% to <60%, and hypoendemic (mf prevalence <20%).

### Predominant vectors.

Three main vectors of onchocerciasis have been recognized in the Venezuelan Amazonian focus of onchocerciasis and are classified based on their respective vector competence (as established by feeding experiments), biting rates on humans (as determined by human-landing catches), and natural infection rates (as measured by fly dissection or molecular methods of parasite detection).^12^^–^^14^ *Simulium guianense* s.l. is the most efficient vector and dominant species in the hyperendemic areas of the southern focus (Figure 1), where it reaches biting densities that vary between 600 and 2,500 bites per person per day. In contrast, *S. oyapockense* s.l. dominates in the hypoendemic areas located in the lowlands and forests of the endemic zone (<200 m above sea level), where it reaches high biting densities (average daily bite rate ∼1,920 bites per person per day). However, unlike *S. guianense* s.l., this species has a buccal structure that destroys mf of *O. volvulus* as it ingests the blood meal; few mf develop into infective larvae as a result, and therefore *S. oyapokense* s.l. has a low efficiency for transmitting onchocerciasis (i.e., a low vector competence). Finally, *S. incrustatum* reaches its greatest abundance between 800 and 1,200 meters above sea level, where it has a biting density of up to 2,000 bites per person per day, contributing to maintaining mesoendemic and hyperendemic levels of *O. volvulus* transmission in the focus (Figure 1). Although it has a mouth structure that also destroys mf, it is less efficient at doing so and is therefore a better vector for transmitting the parasite than *S. oyapockense* s.l.

### History of ivermectin MDA in the Venezuela South Focus.

**Figure 3.**
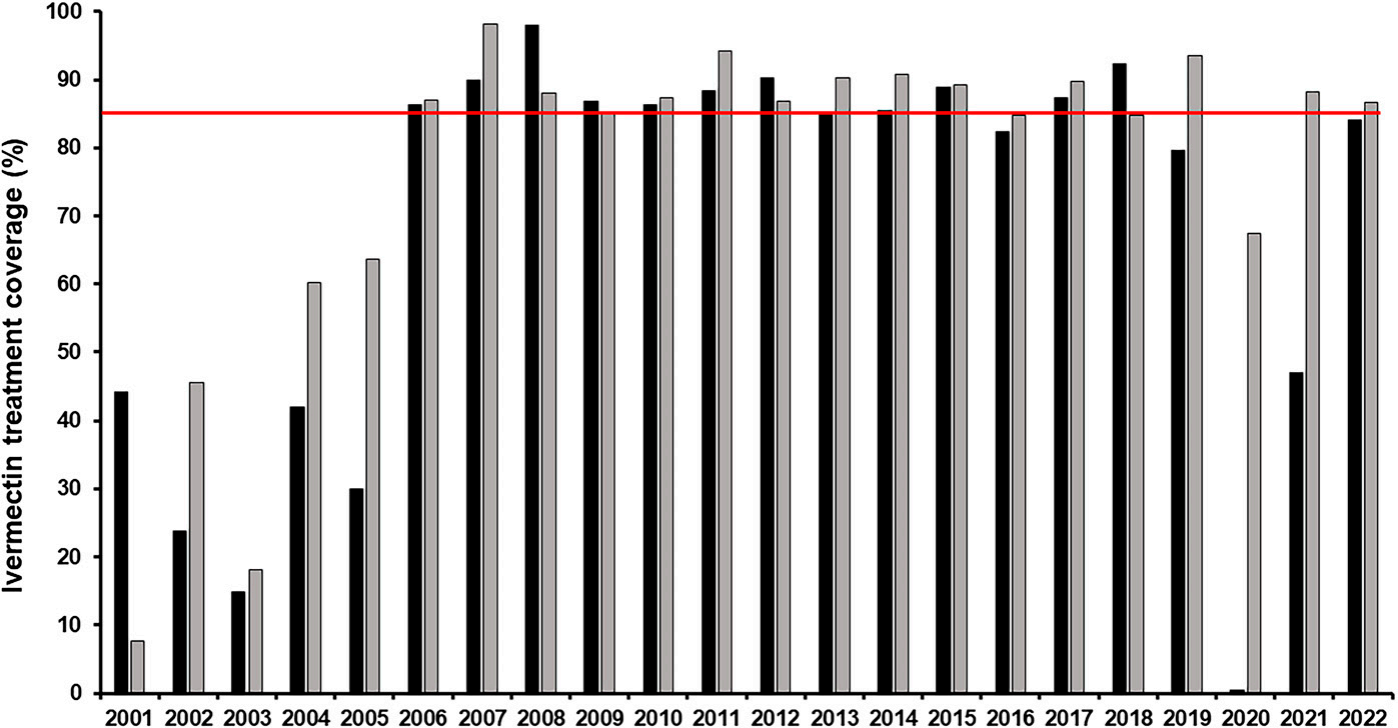
Reported semiannual ivermectin treatment coverage (%) of eligible population in the Venezuela South Focus (2001–2022). The red horizontal line at 85% indicates the target minimum coverage.

**Figure 4.**
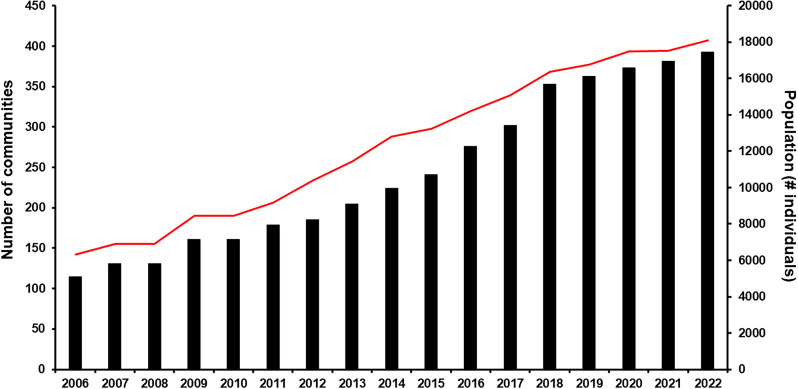
Number of communities (solid bars) and population at risk (gray line) registered in the Venezuela South Focus (2006–2022).

**Figure 5.**
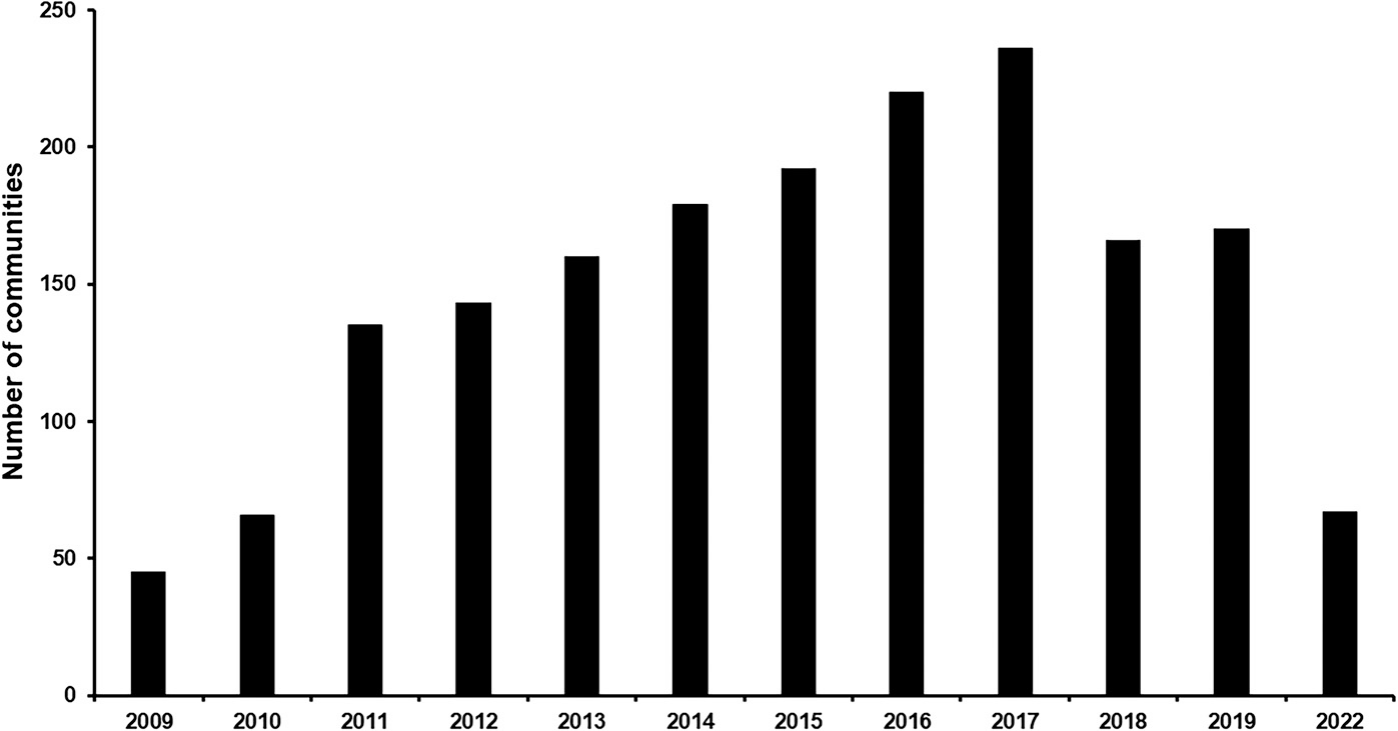
Number of communities in the South Focus receiving four annual rounds of ivermectin treatment (3-month intervals) between 2009 and 2023.

In 2000, the South Focus Onchocerciasis Elimination Program (SFOEP) was reorganized under OEPA’s strategic plan and started ivermectin treatment every 6 months. The ≥85% coverage goal of the eligible population for each treatment round was reached in 2006 throughout the focus, and it was generally sustained until the COVID-19 pandemic (Figure 3). The SFOEP has progressively incorporated communities targeted for ivermectin MDA (Figure 4). Communities occasionally split to form new endemic communities not previously registered by the health system. This has required further on-ground field exploration as well as use of remote-sensing techniques to map and incorporate them into the local health system and the SFOEP.^15^ Communities are reached on foot, by boat, by plane, and by helicopter owing to the difficulties of accessing many of them or to the remoteness of the area. The SFOEP personnel trek through the Yanomami area and navigate rivers in boats and launches, but access to 67% of communities is by air (mainly small planes and helicopters; Figure 2). Often, several flights by small aircraft are required to transport all the personnel and supplies necessary for a single round of MDA. The teams also arrive with the medicines, diagnostic tests, and vaccines required to deliver other primary health care services, such as malaria control and immunizations, to the Yanomami people. Four-times-per-year treatment was launched in a subset of 45 (11%) of the 393 endemic communities in 2009, peaked at 236 (60%) communities in 2017, and then, owing to the challenges of reaching good ivermectin coverage, was reduced to 67 (17%) communities in 2021 (Figure 5). The quarterly treatment began to be administered in selected communities according to the baseline criterion of OEPA priorities, or level of endemicity. That is, hyperendemic communities were a priority. Over time, however, this criterion was not enough to meet the goal of effective coverage in all communities, which led to the need to focus on those communities lagging behind in the number of effective rounds of treatment. Consequently, communities with only 0–10 effective ivermectin treatment rounds were considered at highest risk for ongoing transmission, regardless of endemicity level, and therefore highest programmatic priority for effective ivermectin delivery in every future round. Medium program priority communities were those with 11–19 effective rounds that were believed to still be at risk for ongoing transmission, whereas communities with ≥20 effective ivermectin treatment rounds were thought to have stopped onchocerciasis transmission and were considered lowest priority for the SFOEP.^16^ Here, the quarterly treatment regimen was applied to those communities that had <10 effective treatment rounds. But with experience, the program saw the need to incorporate other variables, in addition to the number of rounds, which would allow it to address logistical and epidemiological aspects at finer spatial scales of the endemic area and be able to meet the objective of advancing in the interruption of onchocerciasis.

### Scorecard Approach.

**Figure 6.**
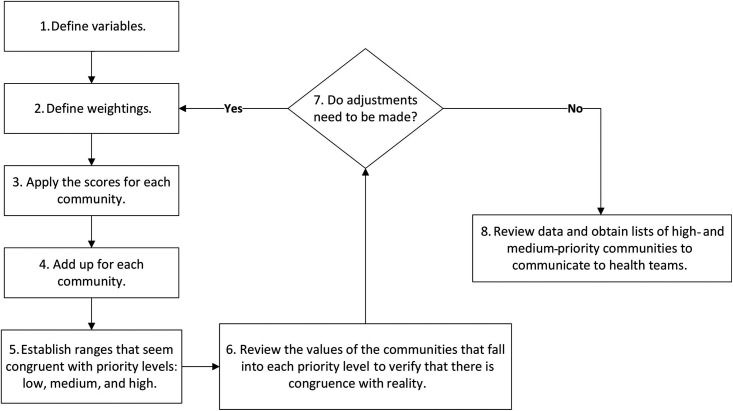
Flowchart of the iterative procedure for the definition, weighing, and final selection of a score for each variable in the SCA of the Venezuelan South Focus of onchocerciasis. SCA = Scorecard Approach.

The purpose of the scorecard was to refine the prioritization of individual community programmatic activities in the Venezuelan South Focus. The design of the SCA for program prioritization began in 2015, when the Venezuelan South Focus and OEPA staff met to determine the variables and weightings that could be summed to generate an overall priority score—the higher the score, the higher the priority. The data for the development of the SCA came from the Venezuelan South Focus records and previous publications and included coverages and epidemiological, entomological, and logistical factors. Senior field staff who had worked for years in this geographic territory provided key input while determining the weighting of variables. From the beginning, it was evident that qualitative and quantitative variables had to be combined, the first coming from the experience of fieldwork and the second being more technical and resulting from treatment coverage and epidemiological evaluations. The main difficulty encountered throughout the selection process was the concordance of variables and their weightings, as well as the collection and summary of the selected data in a single dashboard. This participatory and iterative process is described in the flowchart in Figure 6. The variable and weight definitions and community scoring were a process of continuous discussion that factored in program implementation experiences and made progressive improvements. Step 7 and the collaborative approach to making adjustments (based on personal experience in the field) were vital to reflect commonly perceived realities. The SCA was first applied in 2018 and is reassessed on an annual basis.

## STATISTICAL ANALYSES

The first group of variables consisted of baseline endemicity, dominant vector species, and community access (logistics). The second group contained the number of effective treatment rounds and results of positive or negative prevalence of mf in skin and serology in children under 10 years of age, according to the results of the last epidemiological surveys. The weighting scale was assigned to each possible value for the variables, placing a higher numeric value on the more challenging or worst-case scenario, with intermediate values reflecting a medium-challenge scenario and lower values qualifying a scenario of greater ease in achieving the goals. Lack of data was weighted with a zero value, whereas in those scenarios where the threshold of an indicator had been met, the weight used was −1. For example, when treatment rounds were successfully completed, the weighting was −1, with the total effect of reducing the score in these cases. Each community received a total score. A preliminary observation made it possible to determine and verify the agreement of the total scores with the previously required priority criteria. High-priority communities were targeted for treatment strategies four times a year, focusing resources and operations in these areas. Medium-priority communities also required attention, but less action; that is, some of them needed to continue accumulating effective rounds, whereas others, having passed the threshold of 20 effective rounds, required epidemiological evaluations to determine their current disease transmission status. Table 1 summarizes the selected variables and their weights according to category variation or classification. The first three variables were fixed variables (i.e., the scores given to the communities did not change), and the next three were dynamic variables (i.e., the weightings given to the communities in these variables changed every year according to the program’s actions). Regarding community access and logistics, communities were scored based on how they were reached: by river, by aircraft, with difficulty either by aircraft or river, and by helicopter. Baseline (new community) or regular monitoring entomological surveys in selected sentinel and extra-sentinel communities were the bases for the entomological variable in Table 1. Specifically, all simuliid females landing on two selected human attractants per community were captured with manual aspirators by a team of two collectors during several consecutive collection days.^8^^,^^13^ When entomological information was missing, the dominant biting species was inferred according to the epidemiological landscape of the area.^9^ However, this required future confirmation, hence the term “NA” (data not available) in Table 1.

**Table 1:** Variables and categories used and their respective weights for the development of the SCA in the Venezuela South Focus

Variable	Category	Weight
Baseline Endemicity*	Hypoendemic	1
	Mesoendemic	2
	Hyperendemic	3
Community Access/Logistics	River	1
	Aircraft	2
	Difficulty (either by aircraft or river)	3
	Helicopter	4
Dominant Vector Species	NA^†^	0
	*S. oyapockense* s.l.	1
	*S. incrustatum*	2
	*S. guianense* s.l.	3
Microfilariae (skin)	Zero prevalence	−1
	Prevalence >0	1
	NA^†^	0
Serology	Zero Prevalence	−1
	Prevalence >0	1
	NA^†^	0
Effective Treatment Rounds^‡^	>20	−1
	16–19	1
	11–15	2
	6–10	3
	1–5	4

mf = microfilariae; SCA = Scorecard Approach;* S. guianense = Simulium guianense; S. incrustatum = Simulium incrustatum*; *S. oyapockense = Simulium oyapockense.*

* Hyperendemic is mf prevalence ≥60%; mesoendemic is mf prevalence ≥20% to <60%; hypoendemic is mf prevalence <20%.

^†^ NA = data not available.

^‡^ Effective round means ≥85% ivermectin coverage of eligible population.

Similar to entomological surveys, serological assessments were regularly performed on samples of children 1–10 years of age from selected sentinel and extra-sentinel communities, with the goal of measuring the prevalence of IgG4 antibodies against Ov16, an *O. volvulus* recombinant antigen capable of signaling prepatent infections.^17^ Therefore, this variable in Table 1 was weighted by the prevalence obtained in serological surveys. Similar to the previously mentioned evaluation criteria, when there were no data, it was weighted at zero, at 1 when there was a prevalence greater than zero, and at −1 when the prevalence had reached zero value. The total of effective rounds has been recalculated for each community only since 2006, because as of this year the Program began to steadily reach the 85% target at the focus level, as a reflection of a stronger and more established operability (Figure 3). To obtain the score, effective rounds of two and four times a year were counted equally. In the event that communities split and gave rise to new communities with different names (a very common demographic process among the Yanomami population),^9^ the derived or resulting community was assigned the same number of effective rounds as the community of origin. Finally, the sum total of all factors for each community were calculated.

## RESULTS AND DISCUSSION

Table 2 shows the distribution of the 393 communities in the Venezuela South Focus for each category of SCA variables during 2022. Most of the communities were hyperendemic at baseline (67%); 70.5% of communities were easily accessible either by river or by air, whereas 29.5% were hard-to-reach communities. The dominant vector was known in 89.1% of communities, whereas studies were needed to know the vector species in 10.9% (43) of communities. A majority of the communities lacked parasitological (57.8%) and serological (64.6%) information because the regular monitoring and in-depth epidemiological evaluations had been carried out only in sentinel and extra-sentinel communities (see Table 2). Regarding ivermectin coverage, the program had registered more than 20 effective rounds in 65.6% of communities.

**Table 2:** Distribution of communities by category of variable according to the SCA in the Venezuela South Focus

Variable*	Communities (*N* = 393)	%
Baseline Endemicity^†^		
Hyperendemic	267	67.9
Mesoendemic	48	12.2
Hypoendemic	78	19.8
Community Access and Logistics		
River	131	33.3
Aircraft	146	37.2
With Difficulty (either by aircraft or river)	96	24.4
Helicopter	20	5.1
Dominant Vector Species		
* S. guianense* s.l.	146	37.2
* S. incrustatum*	104	26.5
* S. oyapockense* s.l.	100	25.4
Unknown	43	10.9
Microfilariae in Skin		
Zero Prevalence	23	5.9
Prevalence >0	143	36.4
NA^‡^	227	57.8
Serology		
Zero Prevalence	67	17.0
Prevalence >0	72	18.3
NA^‡^	254	64.6
Effective Treatment Rounds		
>20	258	65.6
16–19	43	10.9
11–15	40	10.2
6–10	30	7.6
0–5	22	5.6

SCA = Scorecard Approach; *S. guianense = Simulium guianense; S. incrustatum = Simulium incrustatum*; *S. oyapockense = Simulium oyapockense.* Regular monitoring and in-depth epidemiological evaluations were carried out in sentinel and extra-sentinel communities that met the following criteria: 1) hyperendemic status, 2) relative ease of accessibility, 3) existence of historical baseline epidemiological data prior to widespread ivermectin distribution; and 4) illustrative of the simuliid species composition of the focus.

* *N* = 393 for 2022.

^†^ Hyperendemic is mf prevalence ≥60%; mesoendemic is mf prevalence ≥20% to <60%; hypoendemic is mf prevalence <20%.

^‡^ NA = data not available.

**Figure 7.**
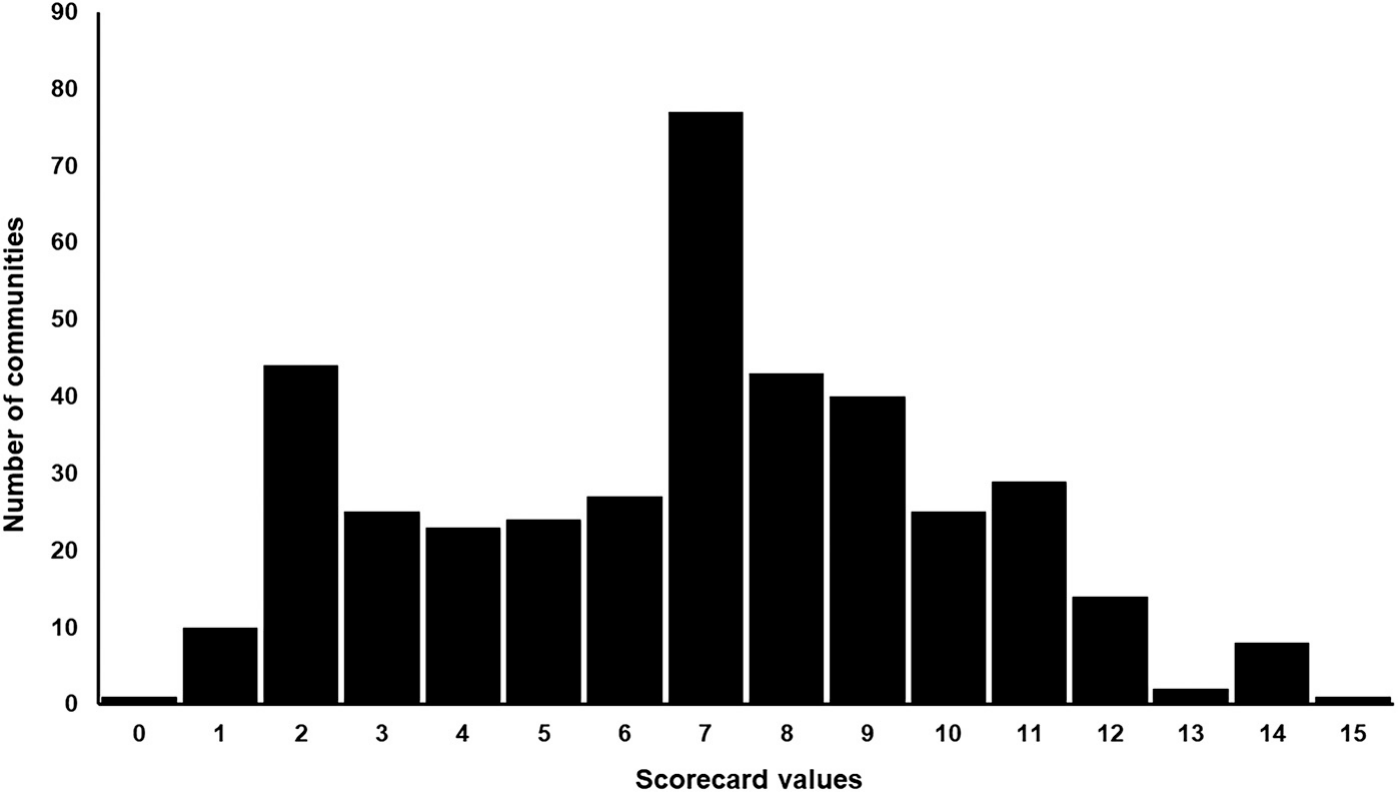
Frequency histogram of SCA. The range is 1–15 (mean ±SE: 6.8 ±0.16). The SCA scores of 11–15 (13.7%) were classified as high priority for program action, 6–10 as medium priority (27.5%), and 0–5 as low priority (58.8%). SCA = Scorecard Approach; SE = standard error.

After the analysis of each variable, the resulting community-level score ranged from 1 to 15 (mean ±standard error: 6.8 ± 0.16). The distribution of scores is shown in Figure 7. We classified SCA scores of 11–15 as high priority for program action, 6–10 as medium priority, and 0–5 as low priority. Table 3 shows that 13.7% of communities were ranked high priority, 27.5% of communities were medium priority, and 58.8% were low priority. For comparison purposes, Supplemental Table 1 shows the resulting prioritization according to the OEPA criteria. Here, 34.3% of the communities were highly or moderately prioritized. By contrast, 41.2% of communities (27 more communities) deserved special attention based on the SCA by 2022, as shown in Table 3. In other words, both schemes showed some differences in the low- and medium-priority levels, but little in the high-priority level. This led us to explore the classification of communities in more detail by taking into account the results of both schemes.

**Table 3:** Treatment priorities for 2022 according to the SCA in the Venezuela South Focus

Variable Level of Priority*							
Spatial Levels	Low	%	Medium	%	High	%	Total
Communities	231	58.8	108	27.5	54	13.7	393
Population at Risk	11,449	63.2	4,536	25.0	2,133	11.8	18,118

* SCA = scorecard approach (11–15 is classified as high priority, 6–10 as medium priority, and 0–5 as low priority).

Table 4 shows the frequency (number of communities) for each classification scheme combination, OEPA versus SCA, better illustrating the relationship and differences between the approaches. Here, we observed 21 communities that, according to OEPA criteria, were medium priority but were high priority for the SCA. When we investigated the characteristics of these communities, we saw that they still had a seropositivity (serology) or prevalence different from zero, in addition to having less than 20 effective rounds. Therefore, these communities required special attention from the Program in relation to a change in the frequency of annual rounds and the need for further epidemiological and serological evaluations to verify the current level of prevalence and parasite transmission. A similar focus should be given to the 108 medium-priority communities according to the SCA (Table 4).

**Table 4:** Changes in community priority for 2022 according to OEPA and SCA schemes in the Venezuela South Focus

OEPA’s Priority	# Communities	SCA’s Priority*	# Communities
Low	258	Low	211
	–	Medium	47
	–	High	0
Medium	83	Low	18
	–	Medium	44
	–	High	21
High	52	Low	2
	–	Medium	17
	–	High	33

OEPA = Onchocerciasis Elimination Program for the Americas; SCA = Scorecard Approach. The SCA’s priority (total # communities): Low = 231, Medium = 108, and High = 54.

* The SCA of 11–15 is classified as high priority, 6–10 as medium priority, and 0–5 as low priority.

**Figure 8.**
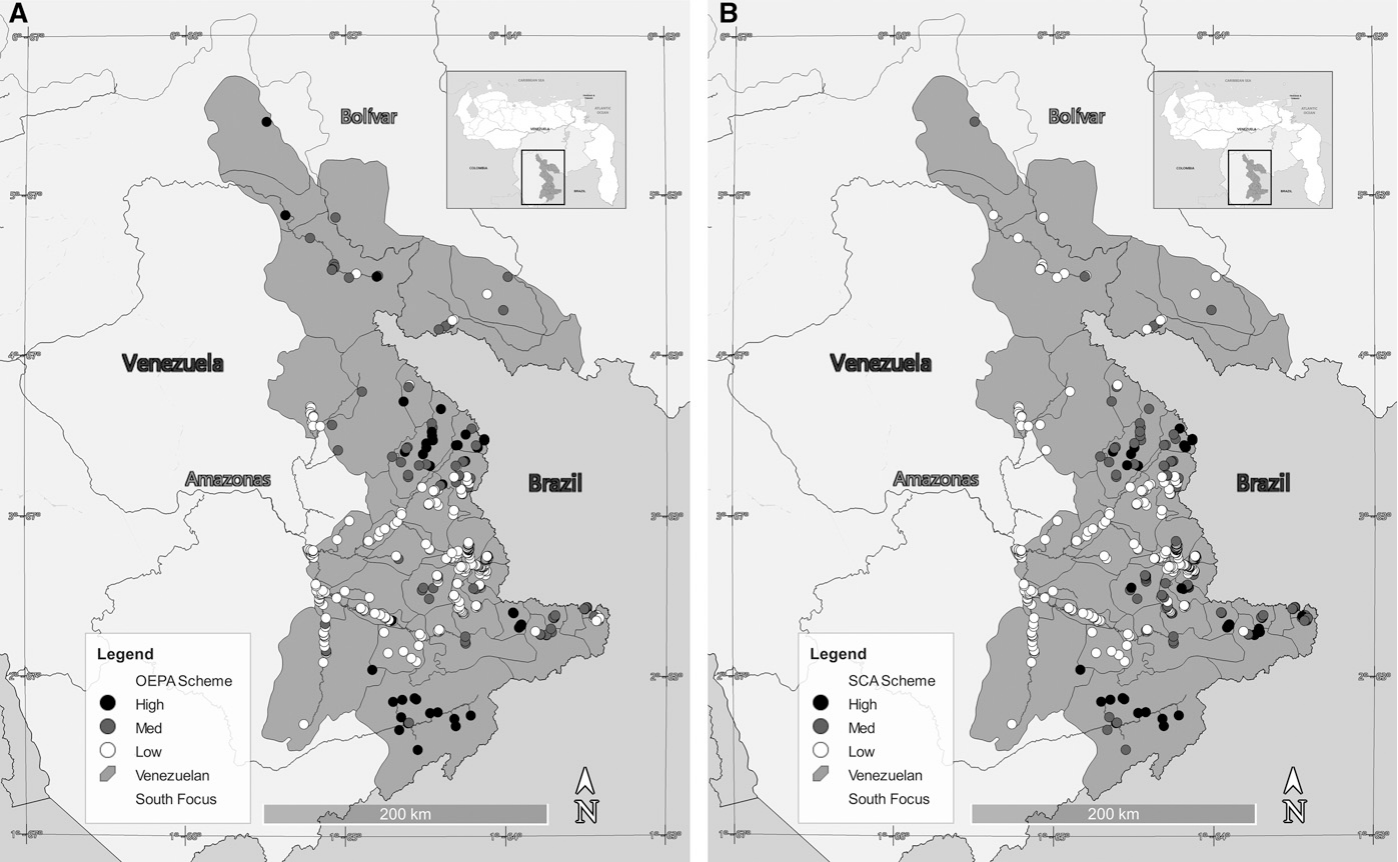
Maps of the onchocerciasis endemic area in the Venezuela South Focus showing number of priority communities for 2022 according to (**A**) OEPA and (**B**) SCA schemes. Med = medium; OEPA = Onchocerciasis Elimination Program for the Americas; SCA = Scorecard Approach.

Another example of the operational nature of SCA is provided by the following examples. In Table 4, there is a group of 17 communities that were classified as high priority according to the number of rounds. However, according to the SCA, they were medium priority. A more detailed analysis revealed that 14 of the 17 communities were difficult to access by air or river, six were communities with the less-efficient vector *S. incrustatum* as the dominant biting species, and three had zero values of seroprevalence. What this indicates is that the number of rounds indicator by itself was not efficiently allocating resources and logistics where they were most needed under the current context of this focus. It is also important to note that within the set of 258 communities with low priority according to the OEPA criteria, none were classified as high priority for the SCA, although 47 were classified as medium priority. Looking at the data, 36 of these communities had a positive prevalence for mf or serology, whereas the remaining 11 lacked indicators. These last communities had high scores in their fixed variables, so despite having more than 20 effective rounds, it was advisable in the short term to carry out epidemiological and serological evaluations to verify the status of *O. volvulus* transmission. Again, the SCA drew attention to different groups of communities than the traditional (OEPA) scheme drew attention to. Although the number of rounds was and has been an important indicator within the SFOEP, the SCA allowed a more complete analysis of multiple scenarios for each community through its scores. Figure 8 depicts two maps of priorities based on the number of rounds (OEPA scheme) versus the SCA scheme to illustrate spatially where to place resources, where to increase the number of rounds, and where to concentrate activities within the Yanomami area. As a complement, Table 5 shows the distribution of the number of communities according to the priority of the SCA at each endemic level. At the hyperendemic level, 46 (13.7%) qualified as high priority and 90 qualified as medium priority. This cluster of 136 communities (see Figure 8) undoubtedly constitutes the most important group for program priority activities in the coming years to achieve elimination of onchocerciasis in the Venezuela South Focus.

**Table 5:** Prioritization based on the SCA results by endemicity level for 2022 in the Venezuela South Focus

SCA Priority*	Baseline Endemicity^†^			Total	%
	Hyperendemic	Mesoendemic	Hypoendemic		
Low	131	32	68	231	58.8
Medium	90	9	9	108	27.5
High	46	7	1	54	13.7

SCA = Scorecard Approach.

* The SCA of 11–15 is classified as high priority, 6–10 as medium priority, and 0–5 as low priority.

^†^ Hyperendemic is mf prevalence ≥60%; mesoendemic is mf prevalence ≥20% to <60%; hypoendemic is mf prevalence <20%.

## CONCLUSION

In summary, here we have presented the development and implementation of a data-driven tool called the SCA for the 393 communities that currently comprise the Venezuela South Focus. This operational strategy attempted to prioritize communities with low ivermectin coverage while taking into account the nature and variation of other epidemiological and logistical variables. During 2022, the SCA categorized 54 (13.7%), 108 (27.5%), and 231 (58.8%) communities as high, medium, and low priority, respectively. The SFOEP continues to implement treatment with ivermectin four times a year in these prioritized communities. Among the main challenges in this endemic area, limited personnel and resources to manage logistics and carry out operations for the program stood out. Despite this, progress continued, as shown by current results from the use of the SCA. This strategy is a continuous process of refinement as well as a useful tool to optimize operations and advance within the SFOEP. One area of needed refinement for the model is the attribution of zero to all unknown parameters. For example, a hyperendemic community with very difficult access, with zero rounds of treatment, and without known mf prevalence, seroprevalence, or vector until the moment of categorization would have scored 10, corresponding to a medium priority. As a result, at the time this article was written, we would have considered an SCA misrepresentation of importance for nine such communities. However, it is good to clarify that communities with medium priority are also the focus of programmatic attention. In addition, missing data in SCA high- and medium-priority communities should be a flag for the program to collect this information as soon as possible. Finally, the SCA should be open to incorporating new variables when necessary, modifying the knowledge we have about a community and incorporating more values into the existing variables for more complete information and guidance, which would allow for improving the indices or values of the score.

The SCA is a methodology derived from similar approaches used recently in the private business sector, healthcare settings, and control programs such as malaria to provide information on areas of strategic importance to guide future planning.^18^ A parallel approach is also being implemented by the Brazilian Ministry of Health in the Brazilian YFA territory (Pereira de Araujo et al., personal communication). To our knowledge, this represents the first time that the SCA has been used in an onchocerciasis elimination context. We hope that this tool can serve as an example and guide program activities in other onchocerciasis-endemic countries as well as for other neglected tropical diseases that may have similar challenges. Despite the usefulness of the standard indicators to monitor the impact of ivermectin established by the WHO guidelines,^6^ the SCA is currently serving as a valuable tool for planning, monitoring, and evaluating onchocerciasis achievement, providing a comprehensive perspective and prioritizing communities with greater healthcare needs on the road to elimination of onchocerciasis in Brazil and Venezuela. The Venezuelan South Focus relies on the collaboration of several national institutions to deliver ivermectin within the focus (e.g., local health authorities and military support for helicopter use); therefore, there was an imminent need for a data-driven tool that could convey which communities still needed to be strongly acted upon to move toward the elimination of onchocerciasis in the focus. This report and its results show that given sufficient commitment and determination by the national and local onchocerciasis program teams and unwavering support by the OEPA, it is possible to attain and sustain high levels of treatment coverage and increased frequency, attesting to the feasibility of interrupting transmission by 2025 in the last endemic corner of onchocerciasis in Latin America, the YFA.

## Supplemental Materials

10.4269/ajtmh.23-0743 Supplemental Materials
