# Supplementary material for: Scorecard Approach to Eliminate Onchocerciasis in Venezuela
Source: Am J Trop Med Hyg. 2024 Jun 11;111(3 Suppl):127–36. doi: 10.4269/ajtmh.23-0743 (PMC11376111; doi:10.4269/ajtmh.23-0743)
Supplement: Supplemental Materials [file tpmd230743.SD1.pdf]

Table 1 (SM)

Treatment priorities for 2022 according to the number of effective (coverage ≥ 85%) MDA rounds delivered (OEPA scheme) in the South Focus of Venezuela

	Level of priority						
	Low	%	Medium	%	High	%	Total
Communities	258	65.6%	83	21.1%	52	13.2%	393
Population at-risk	13553	74.8%	815	4.5%	3750	20.7%	18118

Low-priority: with ≥20 effective ivermectin treatment rounds (transmission suspected to be interrupted)

Medium-priority: with 11-19 effective ivermectin treatment rounds (risk for ongoing transmission)

High-priority: with 0-10 effective ivermectin treatment rounds (highest risk for ongoing transmission)
